# Toward Accurate Position Estimation Using Learning to Prediction Algorithm in Indoor Navigation

**DOI:** 10.3390/s20164410

**Published:** 2020-08-07

**Authors:** Faisal Jamil, Naeem Iqbal, Shabir Ahmad, Do-Hyeun Kim

**Affiliations:** Department of Computer Engineering, Jeju National University, Jejusi 63243, Korea; faisal@jejunu.ac.kr (F.J.); naeemiqbal@jejunu.ac.kr (N.I.); shabir@jejunu.ac.kr (S.A.)

**Keywords:** inertial navigation system, artificial neural network, motion tracking, sensor fusion, indoor navigation system

## Abstract

Internet of Things is advancing, and the augmented role of smart navigation in automating processes is at its vanguard. Smart navigation and location tracking systems are finding increasing use in the area of the mission-critical indoor scenario, logistics, medicine, and security. A demanding emerging area is an Indoor Localization due to the increased fascination towards location-based services. Numerous inertial assessments unit-based indoor localization mechanisms have been suggested in this regard. However, these methods have many shortcomings pertaining to accuracy and consistency. In this study, we propose a novel position estimation system based on learning to the prediction model to address the above challenges. The designed system consists of two modules; learning to prediction module and position estimation using sensor fusion in an indoor environment. The prediction algorithm is attached to the learning module. Moreover, the learning module continuously controls, observes, and enhances the efficiency of the prediction algorithm by evaluating the output and taking into account the exogenous factors that may have an impact on its outcome. On top of that, we reckon a situation where the prediction algorithm can be applied to anticipate the accurate gyroscope and accelerometer reading from the noisy sensor readings. In the designed system, we consider a scenario where the learning module, based on Artificial Neural Network, and Kalman filter are used as a prediction algorithm to predict the actual accelerometer and gyroscope reading from the noisy sensor reading. Moreover, to acquire data, we use the next-generation inertial measurement unit, which contains a 3-axis accelerometer and gyroscope data. Finally, for the performance and accuracy of the proposed system, we carried out numbers of experiments, and we observed that the proposed Kalman filter with learning module performed better than the traditional Kalman filter algorithm in terms of root mean square error metric.

## 1. Introduction

Today, when most of the world is well explored, navigation resides an essential part of our society. Today’s technologies enable us to use the navigation in an entirely new way than our predecessors could. After the invention of smartphones, a vast number of location-based services have been introduced. These location-based services help users to find a way to a certain point of interest. During the last two decades, after the Global Positioning System (GPS) reached it fully operational capacity, the significant of different kinds of location-based services depend on positioning and navigation capabilities have increased tremendously [1,2]. Currently, GPS is recognized as famous for calculating the user’s current location using the satellite. The popular examples of navigation that using GPS are aviation, timing, agriculture, car navigation system and so forth [3]. Even though GPS is considered to be well-known technology for locating the target in an outdoor environment, but it is not feasible for an indoor navigation system as it requires a continuous connection to communicate with satellite [4]. There are many other reasons why GPS will not work in an indoor environment, for example, signal attenuation in an indoor environment because of weak GPS signal, and signal disturbs due to hurdles like steel and concrete walls. The disturbance and hurdles continuously penetrate and block the signal coming from the satellite [5]. Therefore using GPS, it is not reliable to calculate the precise user location in an indoor environment. Hence in consideration of these problems, the GPS is not reliable for indoor positioning systems (IPS) [6,7].

The IPS is a system that used certain information in order to locate the target in an indoor environment. This information includes radio waves, sensors data, WLAN nodes, magnetic field, acoustic signal and so forth [8]. Currently, significant research is being done in the area of indoor localization. However, still, there exist many problems faced by the users due to no standard solution or service for indoor positioning [1]. Nevertheless, many technologies exist that can be used to calculate the position in an indoor environment. The problem with these services is that they were created for other purposes rather than to locate persons or objects, which sometimes make them very unreliable. These issues lead to the development of many miniaturized chips specifically for determining the object or a person in an indoor environment. These chips are called inertial measurement unit (IMU) [9,10].

IMU is an electronic device that is used to measures and detects the body orientation, angular rate, and body-specific force using a combination of accelerometers, gyroscopes, and sometimes magnetometers. During the past several years many IMU has been designed in order to get the precise position estimation in indoor environment [9]. IMU provides a 3-axis sensor, that is, accelerometer, gyroscope, and magnetometer. These sensor data is used to calculate the position of the target in an indoor environment. Double integration is the popular method to calculate the position of the object using accelerometer with respect to time. Similarly, for orientation estimation, Euler angle is used, which includes the information of roll, pitch and yaw using gyroscope data. However, these sensors readings have dynamic noise and bias in their measurements; therefore, we used a different type of filter, for example, Kalman filter, and alpha-beta filter and so forth that are responsible for removing these noise from sensors readings [11,12].

Many solutions have been suggested to predict the position using machine learning (ML). These model uses historical data that reflect the behaviour of the process being modelled. Machine learning techniques for predicting accurate position estimation includes ANNs, adaptive neuro-fuzzy inference systems (ANFIS), support vector machine (SVM), and extreme learning mechanism (ELM). The ANNs method has several advantages over conventional NN as it is easy to use, fast to learn, provide good generalization results, has minimum inaccuracies in training and achieves minimum standard weights. Nowadays, deep learning methods are used in many areas for predictive purposes, such as deep neural networks, deep networks of faith, and recurring neural networks [13,14].

The main contribution of the proposed position estimation based on learning to prediction approaches are followed as:

- The main objective of the proposed system is to get an accurate position estimation by minimizing the error in IMU sensor readings using the prediction algorithm.
- The learning module is based on Artificial Neural Network, and Kalman filter are used as a prediction algorithm to predict the actual accelerometer and gyroscope reading from the noisy sensor reading.
- The learning module continuously controls, observes, and enhances the efficiency of the prediction algorithm by evaluating the output and taking into account the exogenous factors that may have an impact on its outcome.
- In position estimation module, the Kalman filter is used to fuse the IMU data to get noise and drift-free position in an indoor environment.
- Finally, for evaluating system performance, we analyzed the results using the well-known statistical measures such as RMSE, MAD, and MSE. Our proposed system experiments indicate that learning to prediction algorithm improves the system accuracy as compared to tradition prediction algorithm.

Permitting prediction algorithms to encounter ever-changing data or varying surrounding conditions is a demanding job. In this study, we introduce a comprehensive architecture to gain the precision and execution of the prediction module by applying the learning module in indoor navigation. We have used 3-axis sensor values, that is, accelerometer, and gyroscope, which is acquired from the IMU sensor in order to calculate the orientation and position estimation. The design system is comprised of two modules, that is, learning to prediction module and position estimation using sensor fusion in indoor navigation. The learning module is based on ANN and is continuous monitors the prediction algorithm performance by analyzing the output as feedback. The learning module is also responsible for considering the external parameters, (i.e., bias and drifting error) that may affect the outcome of the prediction algorithm. Once, the learning module updates the adjustable settings or commutes the trained model of the prediction algorithm to raise its efficiency regarding prediction accuracy. Similarly, for the learning model, we have used the back-propagation neural network for predicting the accurate parameter to tune the prediction algorithm. The hidden layer comprises of ten neurons, a total of three inputs are assigned to three input layers, and the output layer contains one neuron. The linear and sigmoid functions are employed as activation functions. The structure of the rest of the paper is organized as follows: Section 3 delineates a brief overview of contemporary state-of-the-art approaches; Section 4 encompasses details about the proposed heuristic model. The empirical analysis of the experiments carried out in this study is explained in Section 4, and Section 5 concludes the paper with directions for future work.

## 2. Related Work

**Figure 1:**
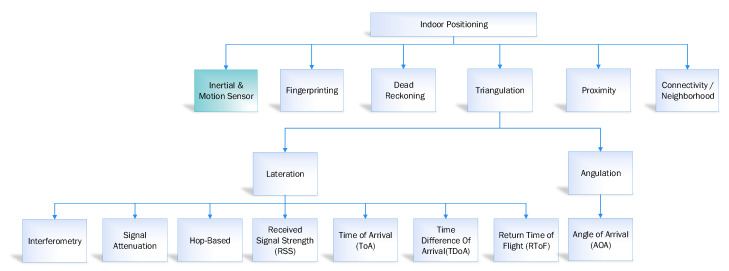
Taxonomy of indoor positioning system.

In navigation, indoor navigation and tracking is a crucial process due to the limited available resources, that is, less GPS signal and satellite availability and so forth. Over the last few years, several location estimation algorithms have been proposed to calculate the distance travelled in indoor and outdoor environments [15,16]. These algorithms are segregated into six categories, that is, fingerprinting, connectivity/neighbourhood, triangulation, inertial and motion sensor, proximity, and dead reckoning [17]. However, in this study, our primary focus is to discuss the IMU-based inertial and motion sensor applications with pros and cons in an indoor navigation system. The overviews of the approaches mentioned above are summarised in Figure 1.

### 2.1. Inertial and Motion Sensor

Inertial and motion sensors are the types of sensors that use information, for example, acceleration, gyroscope, and magnetometer and so forth to calculate the position of the object in an indoor environment. This sensor information like accelerometer is used to calculate the position estimation using double integration method. Thus, the gyroscope tends to determine the orientation using the roll, pitch and yaw. Likewise, the magnetic field direction pertinent to the earth is calculated using the magnetometer. There are many systems proposed during the last few years that uses inertial and motion sensor. The contemporary inertial and motion sensor-based application discussed in the literature below [17].

In Reference [18], the author proposed an algorithm for calculating the orientation of the body using the MEME gyroscope. Moreover, this study also keeps track of sports activity using the IMU and improve the orientation using an extended Kalman filter by removing the uncertainty from the measurement. The performance of the system is measured using the VICON OPTICAL, and it shows that the system is accurate with less root mean square error.

The author of Reference [19] presented a model technique to improve the orientation using the reading output from the inertial measurement unit. A new sensor fusion algorithm MUSE has been implemented for orientation tracking. MUSE is a magnetometer-centric sensor fusion algorithm used orientation tracking. Moreover, this paper also proposed a new sensor fusion method to fully leverage the restriction of human arm movement by shoulder joints and elbow movement.

In Reference [20], the author presented an approach based on artificial neural network. The proposed system is adequate for combining the artificial neural network with the inertial measurement unit in order to get the accurate pedestrian positioning system. The developed system comprised of two possible states, that is, stationary state while the object is not moving regardless of its orientation, and the second is the object is moving equipped with IMU on his body. The further state can also be added to the classification results for the ANN, for example, shaking, jogging, spinning, falling, driving, and flying.

The author of Reference [21] presented an integrated navigation system using a fuzzy logic adaptive Kalman filter (FLAKF). The system is used to overcome the dynamic noise of the accelerometer output and also detect the bias in the sensor reading and resolve the error from the conventional Kalman filter. The main goal of this study is to adjust the weight of the traditional Kalman filter.

In Reference [22], the author presented a distance measuring technique using two methods. The first method for measuring distance is integrating twice the acceleration to get the position estimation. However, in this method, the results are not satisfactory due to exponentially increase in error. Therefore to prevent error, the second method has been implemented, which count the number of steps and angles between legs during movement. The second method uses the accelerometer and gyroscope data to calculate the numbers of steps and angle. The main advantage of the second method is low cost and probability sensor circuit.

### 2.2. Connectivity/Neighborhood

Connectivity/Neighborhood is a method that can be used to analysis of connectivity, that is, numbers of attainable neighbours. In this approach, the numbers of reference points are defined, and they have spatially disseminated the object through which the reference point establishes a connection with the neighbour. In case of signal coverage overlap between the reference point in a suitable way, then the location of the object can be measured using the intersection of all its neighbour’s coverage areas. The accuracy of the presented approach depends on the number of reference points, their distribution and coverage in terms of signal range [17,23].

### 2.3. Proximity

A proximity-based indoor positioning system aims to provide the specific point relative location information to the user whose corresponding point is close proximity. The receiver is used to determines the position of the user when the user is close to the product or an object which is directly connected to the corresponding receiver. The connection to the receiver is based on the signal strength. In case the user is beyond a single receiver then it can be linked with the receiver with the highest signal strength. The proximity-based system can be developed using cell ID (CID), radio frequency identification (RFID), Bluetooth, and infrared radiation (IR) and so forth. These technologies can be used to estimate the location of the users. In the case of CID, a unique number code is used to determine the base transceiver station (BTS). Since the CID of the BTS is received by the mobile users to which they are connected through which the location of the mobile user can be approximated to the proximity of the BTS with the CID information [17,24,25].

### 2.4. Triangulation

The triangulation based indoor position estimation system is based on the geometric properties, which is similar to GPS uses for an outdoor environment. The target location can be identified by several reference points using the angles. The calculation of position estimation using the above-discussed triangulation is called angulation. The common algorithm to measure the angulation is the angle of arrival (AOA). Similarly, to calculate the position using triangulation, we can also use lateration. In the case of lateration the distance of the target location is measured using several reference points. The common algorithms to calculate the lateration are interferometry, hop-based, signal attenuation, received signal strength (RSS), time difference of arrival (TDoA), time of arrival (ToA), and return time of flight (RToF) [17,26,27].

### 2.5. Dead Reckoning

Dead reckoning is the process of calculating the user’s current position using the previously calculated position based on estimated speeds over elapsed time and course. The common example using the dead reckoning are animal navigation, marine navigation, localization of mobile sensor nodes, air navigation, automotive navigation, pedestrian dead reckoning, and directional dead reckoning and so forth [17,27].

### 2.6. Fingerprinting

The fingerprinting algorithm comprises of two steps the training of data and its positioning. The step in training is responsible for constructing a database which contains fingerprints, and in case of step positioning, location estimation is measured using database comparison with existing computing signal strength. In the training process, the database is constructed using the chosen reference point through which the access point signal strength is computed. Finally, each reference point is stored in the database. Similarly, in the positioning step, the signal strength of all the access points is used to measured the target and then compared it with data stored in the database by a deterministic or probabilistic approach. The output of the positioning step is the approximated position of the target. The performance of fingerprinting improves with an expansion in the number of reference points measurements and reference points. The increased number of datum points increases the accuracy as well. Though the training step for fingerprinting is a very burdensome work, and it is demanding in an active indoor condition such as an airport [17,27].

### 2.7. Navigation using Machine Learning Approaches

Nowadays, many machine learning algorithms are used to measure and predict body motion for wearable devices based on IMU Data. Several machine learning systems are working in diverse domain from marketing to medical services [28,29,30,31,32,33,34]. In Reference [35], a fingerprinting based indoor positioning uses a deep neural network to reduce the error in positioning. Similarly, in Reference [36], the author introduced a location-based car park system based on the conventional neural network. This system is used to localize and identify the car in the parking area. In another study, the two indoor localization techniques using machine learning algorithms are used to improve the indoor localization, that is, dead reckoning (DR) and data fusion [37]. In the first method, the DR technique uses an inertial sensor to improve the robustness and continuity of the indoor localization. Similarly, in the second approach, the data fusion approach integrated with machine learning model is used to predict the uncertainty in the wireless-based localization. From the last several decades, ’ many indoor studies have been introduced, which uses the machine learning approaches to predict and track the location of the object in an indoor environment [38]. The contemporary indoor localization integrated with machine learning algorithms uses a different kind of input data such as, inertial sensor data [39], camera data [40], sound data [41] and LiDAR (light detection and ranging) [42]. These input data can be used for several intents, for instance, pass data as an input to the machine learning and get output. Most of the studies output three dimensional location data (x, y, z) axis, angle information [43], angle of arrival [44], distance [45], and object movement status [46].

Table 1 shows the critical analysis of indoor localization based on machine learning algorithms. We concise the comparative analysis into four categories, such as inertial measurement unit data [39,46], radio signal strength [45,47,48,49,50], channel state information [51], and angle of arrival [52].

As below mentioned, Table 1 related to indoor navigation system based on machine learning techniques have many drawbacks in terms of system accuracy and performance. These approaches directly use sensor data as an input to the machine learning algorithm in order to predict and identify the object or location. These sensor reading contains the bias and drifting error which affect the accuracy of the position estimation. However, in the proposed system, we use a prediction algorithm, which is used to minimize the noise in the sensor reading. Moreover, the prediction algorithm is to monitor and control using the artificial neural network to enhance the prediction accuracy of the design system.

As stated above, these systems are not adequately designed for indoor navigation and also have some overcoming in terms of accuracy. To the best understanding of the author, there has been no working tracking scheme for indoor navigation systems appertaining to learning prediction model created as yet.

## 3. Proposed Methodology

The three-axis sensor like accelerometer and gyroscope scope is acquired from next-generation IMU in order to compute the angular velocity ($L_{w}$) and linear acceleration ($L_{a}$) in the proposed position estimation system. The designed system consists of learning to prediction module and the position estimation using sensor fusion in an indoor navigation system. The first module briefly explains the step-by-step working of position estimation using sensor fusion in indoor navigation, and the second module describes the detailed learning to prediction module using ANN and Kalman filter algorithm. Next, we briefly explain the step-by-step working of these models.

### 3.1. Scenario of Position Estimation in Indoor Navigation

**Figure 2:**
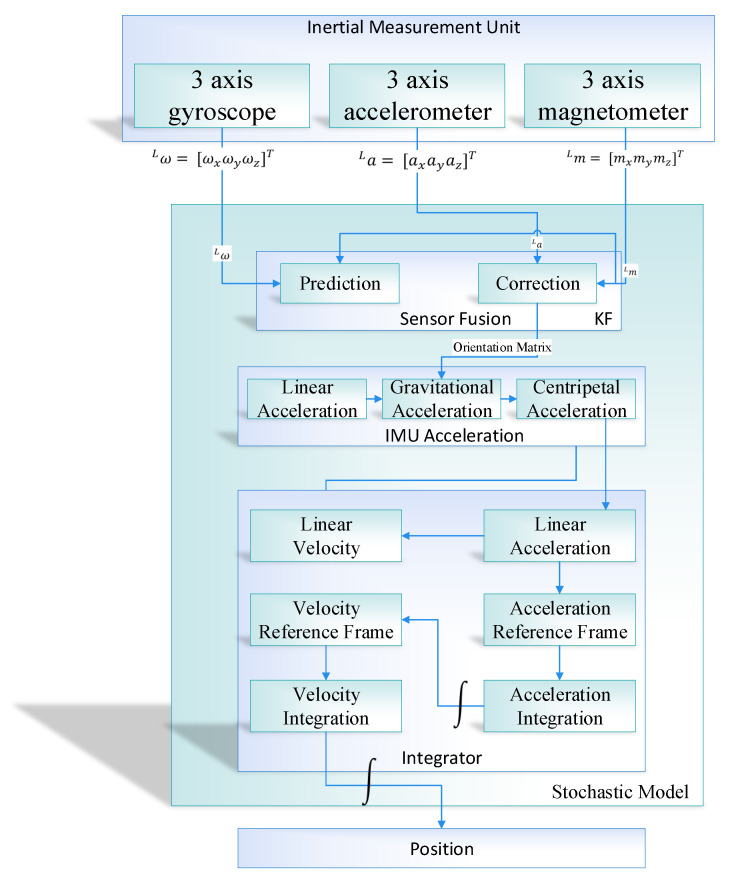
Position Estimation for proposed Indoor Navigation System.

The proposed position estimation system is divided into two modules, that is, the position estimation using sensor fusion and learning to prediction module. The position estimation further divided into four sub-modules (i.e., sensor fusion based on Kalman filter algorithm, IMU acceleration, Integrator, and position estimation) as shown in Figure 2.

The 3-axis output produced by the IMU in the form of a magnetic field vector, angular velocity, and linear acceleration is passed to the sensor fusion module. In the sensor fusion module, the output from the IMU is fuse together using the Kalman filter to get the drift-free and noise-free orientation of the object in an indoor environment. The Kalman filter in the sensor fusion module works as a prediction algorithm, which comprised of two parts, that is, prediction and correction [53,54]. The prediction is calculated by taking the integral of gyroscope measurement and then correct the prediction using the accelerometer and magnetometer readings. The fusion that happens inside the Kalman filter is a probabilistic fusion that combines and correct the inputs based on maximum likelihood. After calculation the drift and noise-free orientation from IMU, the orientation matrix is passed to the IMU acceleration module to remove the gravity and centripetal force. The processed data is further passed to the integrator module in which the velocity is calculated by taking the integral acceleration. Once getting velocity, then take the second integral of velocity to get the position of an object. The integrator is used to performs the mathematical operation of integration concerning time and calculate the velocity and position.

In an indoor environment, the position and orientation of the object are determined by the non-linear matrix. In past researches, accelerometer, magnetometer, and gyroscope’s output gives the orientation estimation, which helps in finding the object orientation. In the case of gyroscope, the complete adjustment could not be calculated at once considering the tendency related to gyroscope readings, as shown in Equation (1). Integrate the angular velocity to get the orientation (roll, pitch, yaw). However, in this case, we have a drifting error.

(1) $$L_{\omega }=L_{\omega_{true}}+b_{g}+v_{g}.$$

Here, $L_{\omega }$ is angular velocity vector in the local sensor frame. $v_{g}$ is a frequency noise, $b_{g}$ is a sensor bias a low-frequency noise we also considered bias as a constant in a small window of time and $L_{\omega_{true}}$ is a true angular velocity that we get from the sensor.

In case of acceleration, assuming there is no motion of the body, the only acceleration measure of gravity is divided by three-component and then uses some trigonometry and gets the roll and pitch with respect to the vertical axis. However, in this case, the accelerometer has a very high-frequency noise, as shown in Equation (2).

(2) $$L_{a}=L_{a_{body}}+L_{g}+b_{a}+v_{a},$$

where $L_{a}$ is the acceleration vector in the local frame, $L_{m_{true}}$ represents the true acceleration due to the motion of the person or object or in other words linear acceleration, $L_{g}$ is the gravitational acceleration, $v_{a}$ is a frequency noise, $b_{a}$ is a sensor bias a low frequency noise we also considered bias as a constant in a small window of time.

**Figure 3:**
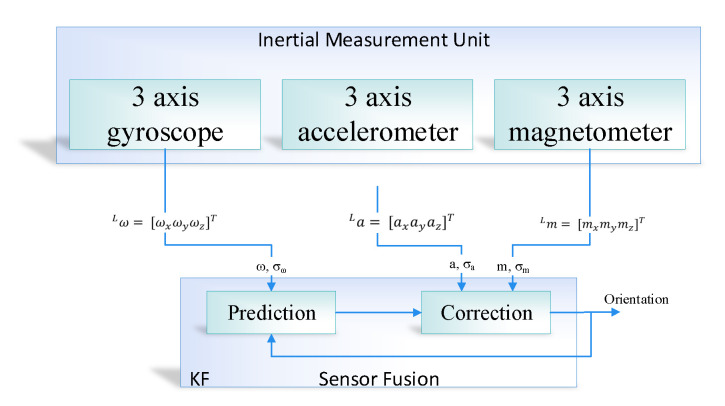
Orientation estimation using sensor fusion based on the Kalman filter algorithm.

In the case of the magnetometer, it is used to calculate the yaw. Therefore, we combine both accelerometer and magnetometer to calculate orientation estimation. However, in this case, we still have noise estimation as represented in Equation (3).

(3) $$L_{m}=L_{m_{true}}+L_{m_{int}}+b_{m}+v_{m},$$

where $b_{m}$ and $v_{m}$ represent the bias and noise. $L_{m_{true}}$ represents the true magnetic field which is the earth magnetic field that is used for heading. $L_{m_{int}}$ is the magnetic disturbances. In order to solve the issue of bias and noise, the idea would be to get the best out of two kinds of estimation. The estimation, which does not have a lot of noise and at the same time does not have the drift. Therefore the best way to get the drift-free and noise-free orientation is to fuse together all the sensors, as shown in Figure 3. In the proposed system, we used the Kalman filter algorithm as a prediction algorithm in order to apply the sensor fusion. In this case, the first step is the prediction step in which we get the data from the IMU sensor and calculate the estimate through the mathematical model and then correct the estimation with measurement (i.e., correction). The Kalman filter comprised of two steps, that is, prediction and correction, in case of orientation estimation of IMU, the prediction is calculated by the integration of gyroscope reading and then correct the measurement through accelerometer and magnetometer reading. The fusion that happens inside the Kalman filter is a probabilistic fusion that combines and corrects the input based on maximum likelihood. In Figure 3, the $\sigma_{\omega }$ is the uncertainty in the gyroscope measurement, and angular velocity is symbolized as ω. Similarly, *a* is represented as acceleration and $\sigma_{a}$ is the uncertainty in acceleration measurement. Finally, the magnetic field is denoted as *m* and $\sigma_{m}$ is the uncertainty in magnetometer measurement.

#### 3.1.1. Sensor Fusion Using Kalman Filter

**Figure 4:**
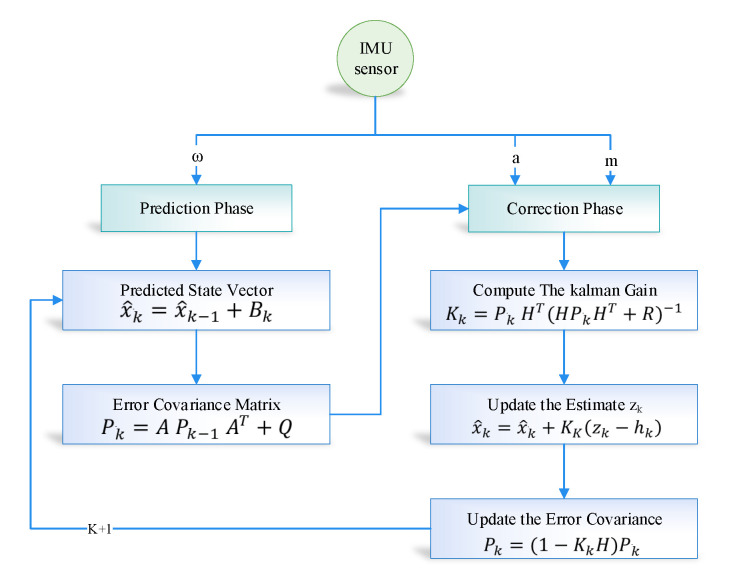
Configuration diagram of Kalman Filter in indoor navigation.

In the study, we used the nonlinear version of the Kalman filter algorithm. The Kalman filter linearized the mean based on the state-space and compute the covariance-based on current state estimation. The Kalman filter is based on a discrete dynamic equation, which can be represented based on two phases; the first phase is the prediction phase, and the second one is the updated measurement phase. In the sensor fusion module of the proposed model, we have used the Kalman filter. The Kalman filter takes the linear acceleration, angular velocity, and magnetic field vector as an input from the sensor and provides the corrected predicted parameter as output. The working flow of the Kalman filter is shown in Figure 4. First, the initial estimation error covariance and initial state are to be determined and calculated using Equations (4) and (5). Besides input, measurement values, process and measurement noise covariance are also considered as an input. Equation (5) helps in finding the gain of Kalman. Both the estimation error covariance and the state estimation based on Equations (7) and (8) for their correctness.

The time update equation of the Kalman filter is:

(4) $${\hat{x}}_{\bar{k}}={\hat{x}}_{k-1}+B_{k}$$

(5) $$P_{\bar{k}}=AP_{k-1}A^{T}+Q.$$

The state update equation of the Kalman filter is:

(6) $$K_{k}=P_{\bar{k}}H^{T}{\left(HP_{\bar{k}}H^{T}+R\right)}^{-1}$$

(7) $${\hat{x}}_{k}={\hat{x}}_{\bar{k}}+k_{k}\left(z_{k}-h_{k}\right)$$

(8) $$P_{k}=\left(I-K_{k}H\right)P_{\bar{k}},$$

where the predicted state-vector is represented as ${\hat{x}}_{\bar{k}}$, which includes 3-axis coordinates along with the heading using the proposed sensor fusion technique. $B_{k}$ is the state space model matrix. Furthermore, $P_{\bar{k}}$ is the error covariance matrix which is comprised of two parts—(i) predicted error noise symbolized as (Q); (ii) State matrix which is denoted as A. The $P_{k}$ value is modified at every iteration using three parameters. The parameters are predicted error covariance matrix ($P_{\bar{k}}$), obtained Kalman-Gain ($K_{k}$), and updates the measurements (*H*).

#### 3.1.2. IMU Acceleration

**Figure 5:**
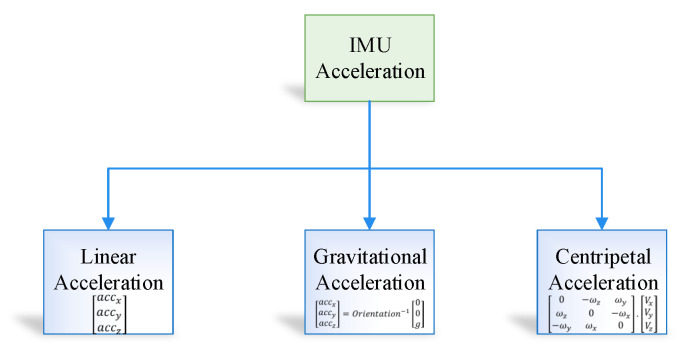
Inertial measurement unit (IMU) accelerometer output.

The output from the IMU accelerometer is divided into three component, that is, IMU acceleration, gravity correction, centripetal force correction, as shown in Figure 5.

In the proposed position estimation system, we use linear acceleration in order to obtain the position in an indoor environment; therefore, it is essential to remove both centripetal and gravitational force from the sensor data. The IMU fixed frame contains gravity acceleration, which can be formulated in Equation (9)

(9) $$\left[\begin{array}{c} acc_{x_{gravity}}\\ acc_{y_{gravity}}\\ acc_{z_{gravity}} \end{array}\right]\left(t\right)=Orientation^{-1}\left(t\right)\left[\begin{array}{c} 0\\ 0\\ g \end{array}\right],$$

where gravitational acceleration magnitude is symbolized as g and acceleration vector with respect to earth fixed frame is denoted by $\left[\begin{array}{c} 0\\ 0\\ g \end{array}\right]$. Similarly gravity acceleration vector with respect to IMU fixed frame symbolized as $\left[\begin{array}{c} acc_{x_{gravity}}\\ acc_{y_{gravity}}\\ acc_{z_{gravity}} \end{array}\right]$.

The rotation of the object can take place in two ways—(i) the object rotation around a point in the space and (ii) the object rotation around itself. Therefore, in the proposed system the rotation of the object is calculated using the Equation (10):

(10) $$\left[\begin{array}{c} acc_{x_{centripetal}}\\ acc_{y_{centripetal}}\\ acc_{z_{centripetal}} \end{array}\right]=\left[\begin{array}{ccc} 0 & -\omega_{z} & \omega_{y}\\ \omega_{z} & 0 & -\omega_{x}\\ -\omega_{y} & \omega_{x} & 0 \end{array}\right].\left[\begin{array}{c} V_{x}\\ V_{y}\\ V_{z} \end{array}\right].$$

The centripetal force is defined as the cross product of the linear velocity and angular velocity.

#### 3.1.3. Integrator Module

Integrator is used to performs the mathematical operation of integration with respect to time and calculate the velocity and position from the linear acceleration [55]. The integrator module is based on the following steps. In the first step, we take the integral of acceleration measurement to get the velocity of the object in an indoor environment, as shown in Equation (12). In Equation (11), a denotes the acceleration measurement taken from the IMU sensor.

(11) a=constant

(12) $$v=\int adt=v_{o}+at.$$

In the second step, we take the integral of the computed velocity to get the position of the object in indoor environment. The Equations (12)–(14) demonstrate the second integration, where *y* donates position, velocity is denoted as $v_{0}$, *t* represents time and *a* is the acceleration.

(13) y=∫vdt

(14) $$y=\int (v_{o}+at)dt$$

(15) $$y=y_{o}+v_{o}t+\frac{1}{2}at^{2}.$$

### 3.2. Learning to Prediction Model

**Figure 6:**
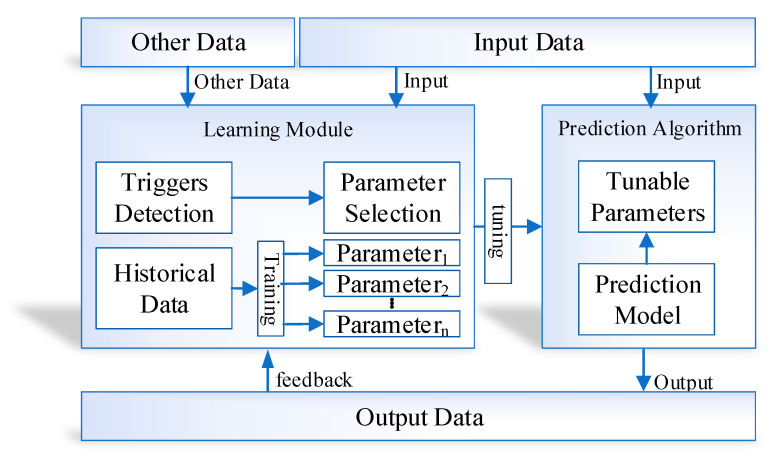
Conceptual view of the proposed learning to prediction model in indoor navigation system.

The proposed learning to prediction model is classified with a couple of modules, that is, the prediction algorithm and learning module. Traditionally, the historical data are used to train the prediction algorithm so that the relationship and hidden pattern can be learned among the output parameters and the input parameters. Afterwards, the output of any given input data is predicted using the trained model. The performance of the prediction algorithm depends upon a couple of things. The training data conditions are as same as the application scenario data and the input data. Nevertheless, none of the current prognostication algorithms adopts model well enough to train dynamic input states. Therefore, to vanquish the existing studies’ limitations, we design a novel learning to a prediction model for the proposed indoor navigation system, as illustrated in Figure 6.

In the proposed learning to prediction model, the prediction algorithm is tuned using the learning module in order to improve the accuracy and performance of the prediction algorithm. Furthermore, the learning module continuously evaluates the performance of the prediction algorithm by receiving the output as feedback. The external parameter is also considered by the learning module that may create an impact on prediction algorithms in terms of performance. After monitoring the current output and external factors, the learning module updates the tunable parameters of the prediction algorithm or upgrade the complete trained model in the prediction algorithm to improve the prediction accuracy.

**Figure 7:**
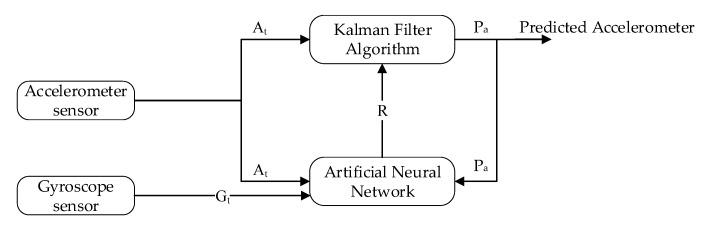
Block diagram of Accelerometer prediction using learning to prediction model.

**Figure 8:**
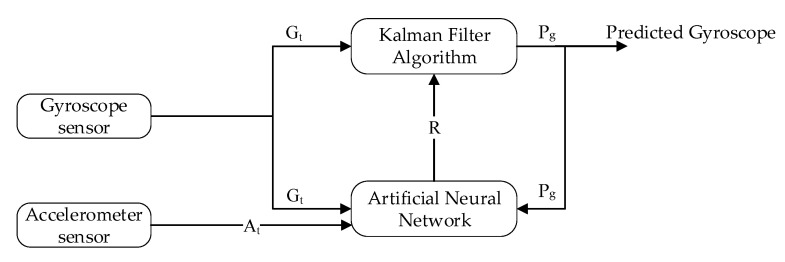
Block diagram of Gyroscope prediction using learning to prediction model.

The design learning to prediction model comprised of two-part, that is, learning model and the prediction model. The learning model is based on ANN, and the prediction model uses a Kalman filter algorithm as a prediction algorithm, as demonstrated in Figure 7 and Figure 8. In Figure 7, the Kalman filter algorithm is used to predict the actual accelerometer readings from the noisy accelerometer sensor readings, which are heavily influenced by the gyro bias. Traditionally the Kalman filter algorithm does not require historical data for prediction, but it only requires the previous state to predict the actual state of the system. Similarly, in the case of the learning module, we have used the feed-forward back propagation neural network (FFBPNN) to tune the prediction algorithm. The learning module takes three inputs, that is, accelerometer reading, gyroscope reading, and the Kalman filter predicted reading as feedback. The accelerometer sensor reading $A_{t}$ is passed to Kalman filter as input at time *t*, and the output from the Kalman filter is the predicted accelerometer sensor reading $P_{a}$ without noise. Noise in accelerometer sensor readings is due to the gyro bias, which is $G_{t}$.

Similarly, in Figure 8, the gyroscope reading $G_{t}$ is predicted using the Kalman filter algorithm, and the output is predicted gyroscope sensor reading $P_{g}$ without noise. Noise in the gyroscope is due to the impact of accelerometer value.

In the intended learning to prediction model, the tunable parameter can control the prediction algorithm performance, which is Kalman, gain *K*. To intelligently update the gain *K* after every iteration, the estimated error in sensors’ reading *R* and the covariance matrix *P* are used. The next subsection describes the Kalman filter in detailed.

### 3.3. Kalman Filter Algorithm

**Figure 9:**
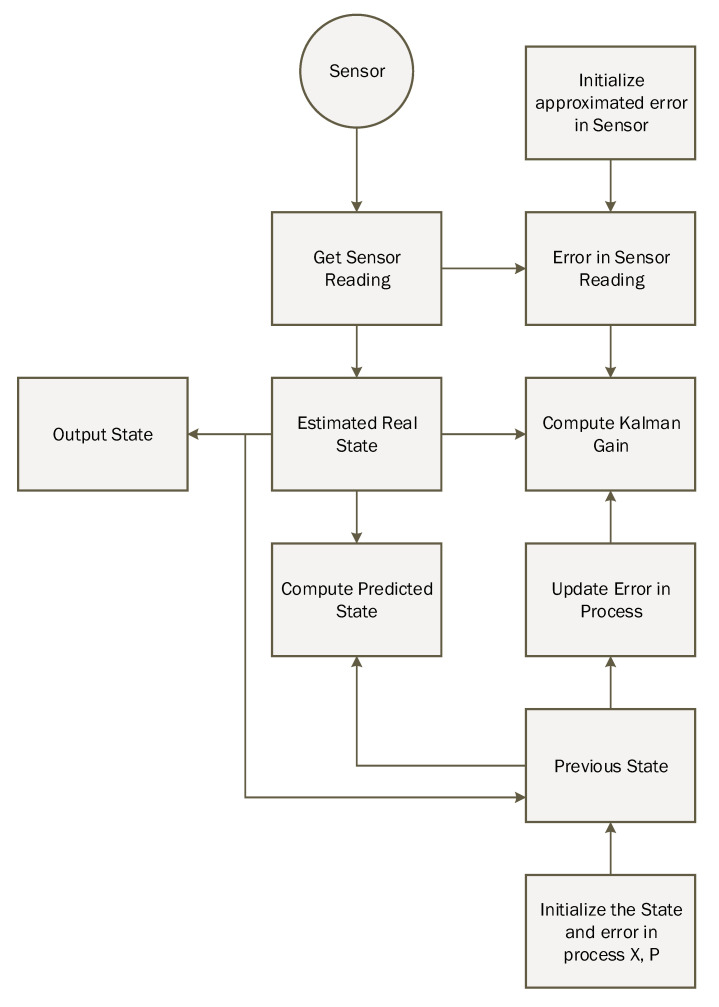
Working of the Kalman filter algorithm.

The requirement of historical data in the Kalman filter is not necessary because of its lightweight prediction. For intelligent prediction, the Kalman filter only required previous state information in order to predict the actual state of the system. *K*, which is also known as Kalman gain, is the necessary parameter that is updated based on the situation to control weights given to the system’s own predicted state or sensor readings. The detailed working of the Kalman filter is illustrated in Figure 9.

In every environment, there is a noise factor which creates a serious impact on sensor readings in that environment. In the proposed methodology, we considered an accelerometer and gyroscope sensor reading having noise, and let us considered $P_{a}$ and $P_{g}$ is the accelerometer and gyroscope at time *t*. The internal prediction regarding system state is based on Kalman filter, that is, estimated accelerometer $P_{a+1}$ at time t+1. Afterwards, we describe the step by step process of the Kalman filter algorithm, that is how it removes the noise from sensor data.

The first step is to compute the predicted sensor reading, that is, accelerometer and gyroscope from the previously estimated value using Equation (16).

(16) $$S_{p}=A.AG_{t-1}+B.u_{t},$$

where $S_{p}$ is the internally predicted sensor reading, that is, accelerometer and gyroscope, the state transition and control matrix is denoted as A and B receptively. $AG_{t-1}$ is the predicted accelerometer and gyroscope at time t−1 that is previously computed and control vector denotes as $u_{t}$.

The uncertainty in the internally predicted sensor reading, that is, accelerometer and gyroscope is calculated using the covariance factor which is computed using Equation (17)

(17) $$P_{pred}=A.P_{t-1}.A^{T}+Q,$$

where the estimated error in the process is symbolized as *Q*, and the old value of covariance is denoted as $P_{t-1}$. *A* is the state transition matrix, and $A^{T}$ represents the transpose of the state transition matrix.

Equation (18) compute the internal system estimate of the next state and updating covariance based on Kalman gain.

(18) $$K=\frac{P_{pred}.H^{T}}{H.P_{pre}.H^{T}+R},$$

where an estimated error in the readings is denoted as *R*, *H* and $H^{T}$ are the observation matrix and its transpose.

We consider a scenario in which current reading obtained form the accelerometer sensor at time *t* is denoted as $A_{t}$. Similarly, in case of gyroscope, current reading, which is symbolized as $G_{t}$ is passed to the Kalman filter. Afterwards, the predicted accelerometer $P_{a}$ and predicted gyroscope $P_{g}$ given by the Kalman filter is computed using the Equation (19).

(19) $$P_{a}=P_{a_{pred}}+K\left(G_{t}-H.P_{a_{pred}}\right)$$

(20) $$P_{g}=P_{g_{pred}}+K\left(A_{t}-H.P_{g_{pred}}\right).$$

**Figure 10:**
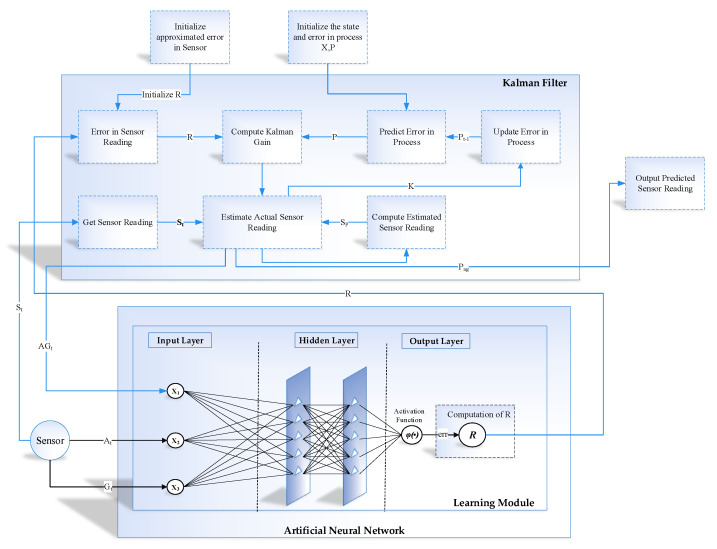
Position Estimation based on learning to prediction.

From Figure 10, we calculated the predicted sensor reading using Equation (21)

(21) $$AG_{t}=AG_{pred}+K\left(S_{t}-H.AG_{pred}\right).$$

In Equation (22), the covariance for the next iteration is finally updated as below

(22) $$P_{t}=\left(I-K.H\right)P_{pred}.$$

### 3.4. ANN-Based Learning to Prediction for Kalman Filter

The traditional Kalman filter works fine when there is no changing in the estimated error in the sensor. However, if the change in the estimated error occurred due to some external factor, then we need to update the value of *R*, which is the estimated error in the measurement as shown in Figure 9. In the designed system, we considered a scenario where accelerometer data is affected due to gyro bias, and similarly, accelerometer value causes the noise in the gyroscope. The traditional Kalman filter fails to predict the actual accelerometer and gyroscope under these dynamic conditions when the value of sensor reading changed due to external factors. Figure 10 shows the complete functioning layout of the intended learning to prediction model. The three inputs of the learning module are the previously predicted sensor value by Kalman filter algorithm, acceleration and gyroscope. The expected error is the output in the sensor reading, which is moreover divided by the fixed constituent denoted as *F* to calculate the estimated error in the sensor reading that is *R*. Afterwards, the Kalman filter takes the updated value of *R* as input and adjusting the Kalman gain *K* by appropriately tuning its prediction accuracy. The intended model predicts the actual accelerometer and dynamic error rate reading from the noisy sensor reading of gyroscope.

## 4. Experimental Results and Discussion

### 4.1. Development Environment

The development environment of the proposed work is categorized into two models, i.e., learning to prediction model and position estimation using the sensor fusion algorithm. In the case of the stochastic model, we have used the next-generation inertial measurement unit(NGIMU), the next generation inertial measurement unit designed for data acquisition with the onboard sensor and data processing algorithm. The on-board sensor includes 3-axis accelerometer, gyroscope, and magnetometer sensor, which is further used to calculate the position in an indoor environment. The characteristic of NGIMU is mentioned in Table 2.

The next generation inertial measurement unit sensor was used to acquired data for inertial navigation in an indoor environment. The data were taken while the object walked from corridor to room number D242 in building 4 of Jeju National University, Republic of Korea. The sample data were acquired for a time duration of approximately 1 minute in which the first starting 10 sec remained inactive so that the algorithm could congregate in a stable state. The tool and technologies for developing the proposed stochastic model are summarized in Table 3. Similarly, for the learning to prediction model, we used an ANN for tuning the prediction algorithm in order to enhance the accuracy of the prediction algorithm. The detailed summary of the development environment for learning to prediction model is mentioned in Table 4.

All the implementation and experiments of the proposed system were carried out on Window 10 64 bit with 8GB memory and Intel(R) Core i5-8500 @ 3.00GHz processor. Furthermore, for development, we used MATLAB R2018a and the NGIMU application programming interface (API) to acquire the sensor data.

### 4.2. Implementation

The proposed system is developed in order to evaluate the performance of the prediction algorithm, that is, the Kalman filter with the learning module. The experiment was performed on the real dataset taken in the engineering building of Jeju national university. At the start, the data were loaded into the application through NGIMU API. The data has ten inputs, that is, 3-axis accelerometer, 3-axis gyroscope, 3-axis magnetometer and time at which the data is taken. Afterwards, we calculated the orientation using sensor fusion based on the Kalman filter. The orientation matrix was further processed by removing the gravitational and centripetal forces and finally applied double integration to compute the position of an object in an indoor environment. Furthermore, the RMSE value of IMU sensor reading was computed by comparing its values with the actual IMU data such as the accelerometer and gyroscope sensor values. The RMSE for the IMU sensor reading was recorded as 5.25, which is considered very high.

Moreover, we used a Kalman filter algorithm to forecast the actual sensor reading (i.e., accelerometer and gyroscope) form the noisy sensor reading. The develop interface provides manual training of the internal parameter of the Kalman filter, that is, the estimated error in measurement (R). Multiple experiments were carried out with different values of *R* in order to evaluate the performance of the proposed system. The RMSE of the predicted accelerometer and gyroscope was 2.30 at R=20. The predicted RMSE was better than the RMSE of sensor readings, that is, a 55% reduction of error.

**Figure 11:**
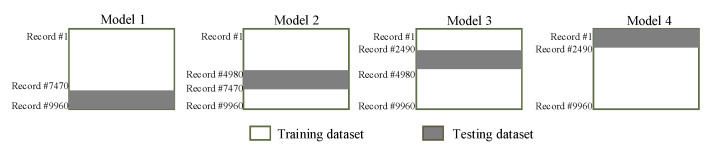
n-fold cross validation.

In the learning to prediction module, we had to use the ANN algorithm, which is used to enhance the accuracy of the prediction algorithm. The ANN algorithm was comprised of three neurons as an input layer and one neuron as a layer (i.e., accelerometer, gyroscope and Kalman filter predicted reading) and predicting the error in the sensor reading, respectively. Furthermore, we used n-fold cross-validation in order to avoid bias in the training process. For this purpose, we split the dataset into four equal subsets (i.e., 2490 samples in each subset) as shown in Figure 11. According to 4-fold validation, 75% of the dataset was used for training, and other 25% was used for testing the ANN algorithm. Furthermore, in the proposed system, we used 100 epochs which were used for training the ANN algorithm. In the ANN-based learning module, the data normalization was done using Equation (23).

(23) $$\tilde{d_{i}}=\frac{d_{i}-d_{min}}{d_{max}-d_{min}},$$

where $\hat{d_{i}}$ is the normalized value for the $i_{th}$ position of the input and output parameters, that is, accelerometer, gyroscope and predicted sensor data. The maximum and minimum value for each parameter in the dataset is denoted by $d_{min}$ and $d_{max}$. Traditionally, in ANN training is done using normalized data, therefore in order to compute the predicted error we de-normalized the output data of the neural network using Equation (24).

(24) $$er_{i}={\tilde{er}}_{i}x\left(er_{max}-er_{min}\right)+er_{min}$$

Furthermore, the proposed model accuracy was evaluated using three different matrices such as mean absolute deviation (MAD), root mean squared error (RMSE), and mean square error (MSE) as shown in Equations (25)–(27).

(25) $$MAD=\sum_{n}^{i+1}\left|T_{i}-\hat{P_{i}}\right|$$

(26) $$MAE=\sum_{n}^{i+1}{\left(T_{i}-\hat{P_{i}}\right)}^{2}$$

(27) $$RMSE=\sqrt{\frac{\sum_{i+1}^{n}{\left(T_{i}-\hat{P_{i}}\right)}^{2}}{n}},$$

where total observation is denoted as *n*, the target value is represented as *T*, and $\hat{P}$ indicates the estimated value.

### 4.3. Results and Discussion

**Figure 12:**
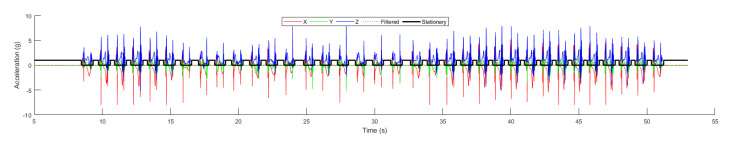
Acceleration.

The open-source NGIMU was used to acquired data in order to calculate the object position in an indoor environment. Moreover, for analyzing the proposed system performance, we compared the result predicted by the conventional Kalman filter algorithm with the learning to prediction model. In Figure 12, the raw accelerometer data is shown, which was acquired from the next-generation inertial measurement unit along with the time at which the data were taken. The 3-axis representation of the accelerometer data is denoted by x, y, and z. The dotted line represents the filtered data using a Butterworth filter based on the defined cut-off frequency, and the solid black line shows the stationary data, which is the magnitude of 3-axis acceleration. The stationary data represent the state of the object if the magnitude is less than 0.05; the object state is stationary; otherwise, the object is moving.

**Figure 13:**
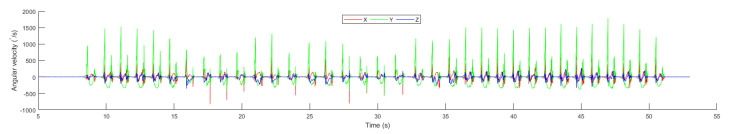
Gyroscope.

Figure 13 shows the value of the gyroscope, through which we calculated the value of the angular velocity of the moving object. The 3-axis gyroscope is represented by x,y, and z. The first integration of the angular velocity with respect to time leads to Euler angle, which is required to define the orientation of the object. The Euler angle is usually used to calculate the Roll, Pitch, and Yaw. The angular velocity is the rate of change in prescription of the object moving over time. The formula of angular velocity is mentioned in Equation (28).

(28) $$\omega =\frac{\theta_{f}-\theta_{i}}{t},$$

where $\theta_{f}$ and $\theta_{i}$ denotes the final angle and the initial angle of the object. The change of angle is denoted by Δθ, and finally t represents the time.

**Figure 14:**
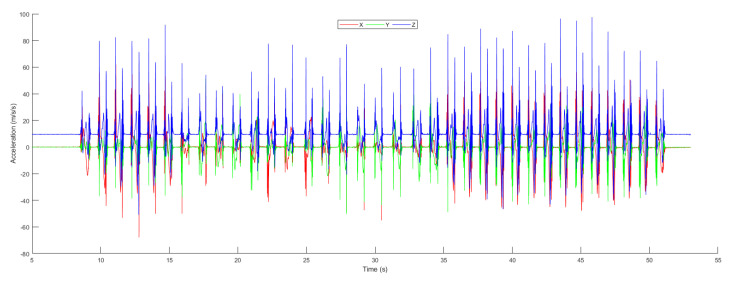
Acceleration $m/s^{2}$.

Figure 14 illustrated the acceleration $m/s^{2}$ of the object in an indoor environment. The acceleration $m/s^{2}$ of the object is measured as the rate of velocity over time and is calculated using the formula mentioned in Equation (29)

(29) $$a=\frac{\Delta v}{t},$$

where Δv denotes the change in velocity, a represent the acceleration in $m/s^{2}$ and time is denoted by t.

**Figure 15:**
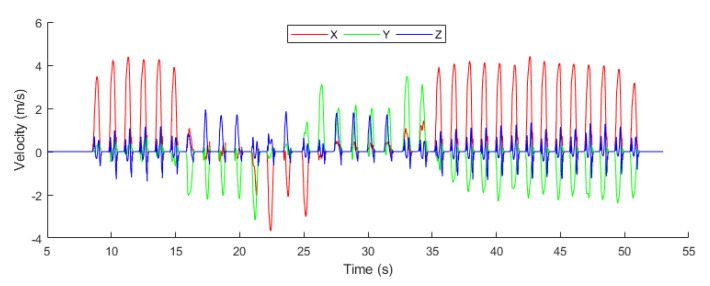
Velocity.

The velocity of the object was measured as to how fast the object is moving in an indoor environment. Figure 15 illustrated the 3-axis velocity of the object, which is calculated using the formula mentioned in Equation (30).

(30) $$v=\frac{\Delta x}{t},$$

where Δx represents the change in position of the object within an indoor environment, *v* denotes velocity and *t* represents the time at which the object changes its position.

**Figure 16:**
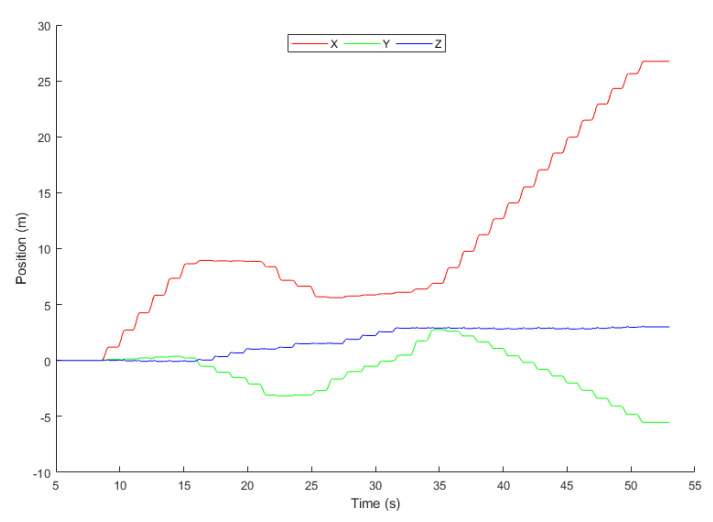
Position.

In Figure 16, the 3-axis position of the object is represented using a 2-dimensional graph where x, y, and z represent the axis.

**Figure 17:**
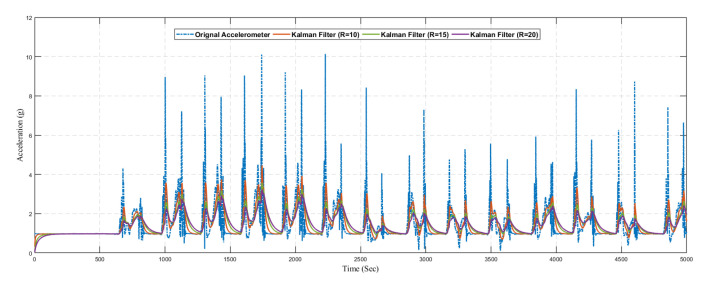
Position.

In Figure 17, we present the predicted result of the accelerometer sensor using the traditional Kalman filter without the learning model. We compared the original sensing data with different values of *R*. The optimal value of *R* is based on the dataset, and it is not fixed. Hence it is difficult to find the optimal value of *R* manually, so we considered different values of *R*. Also from the graph, it can be seen that the Kalman filter prediction accuracy changed with changing the values of *R*

**Figure 18:**
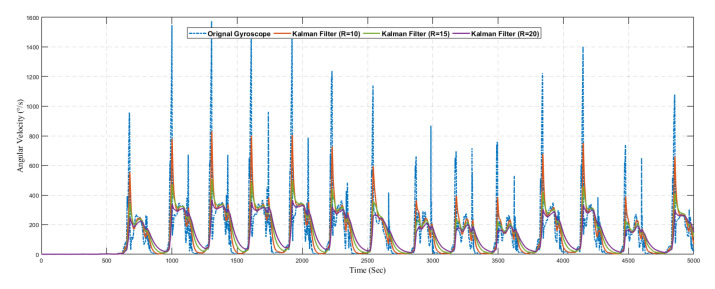
Position.

Similarly, Figure 18 shows the result of predicted angular velocity using a conventional Kalman filter without the learning model. The graph represents the variation in prediction results by varying the value of *R* in the Kalman filter configuration. The gyroscope sensor reading is predicted using three different configurations as summarized in Table 5.

**Figure 19:**
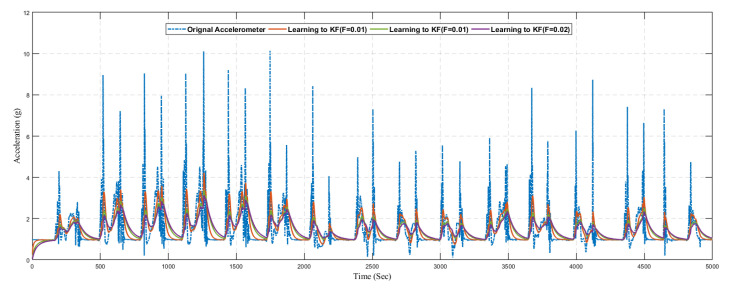
Prediction of acceleration using learning to prediction model with selected *F* values.

**Figure 20:**
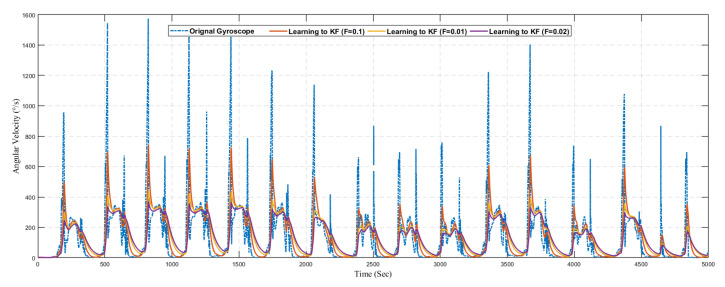
Prediction of gyroscope using learning to prediction model with selected *F* values.

Next, we present the results of the proposed learning to prediction for both accelerometer and gyroscope sensors readings, as illustrated in Figure 19 and Figure 20. We used the ANN trained model in order to improve the performance by tuning its *R* parameter. The predicted error rate update the R for the Kalman filter algorithm based on *R*; we choose the suitable value for *F*, also called the error factor using Equation (31).

(31) $$R=\frac{er_{i}}{F},$$

where proportionality constant also called as error factor denoted as *F*.

**Figure 21:**
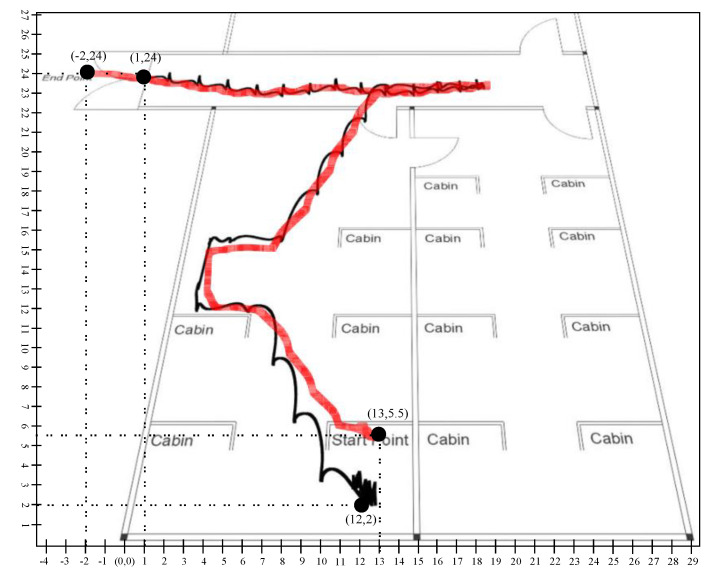
Scenario 1: Person tracking in indoor environment.

**Figure 22:**
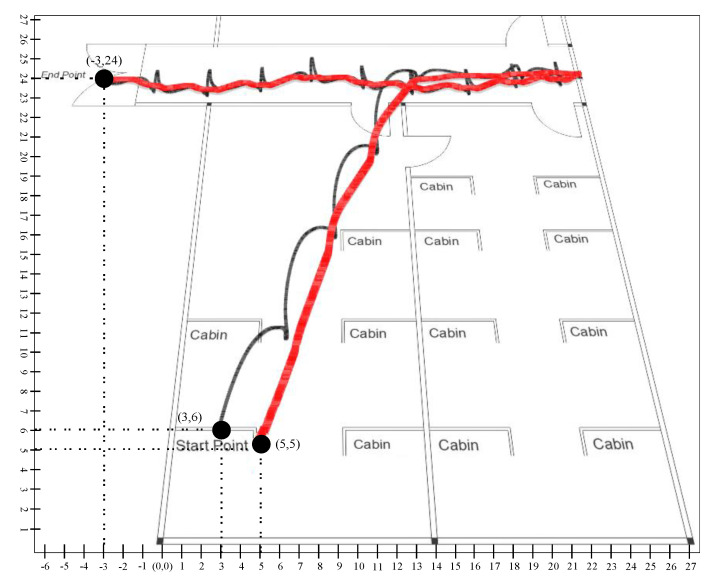
Scenario 2: Person tracking in indoor environment.

The graph shows the position data for 60 s in which the first 10 s represents the stationary state. If we investigated the position plot, then we came to know that the proposed system significantly reduced the drift and error from the sensing data. Figure 21 and Figure 22 illustrated the trajectory of a person walking in room no D242 toward the main corridor. In both scenarios, the person starts walking from the starting point where the first 10 s remained stationary so that the algorithm could converge on a stable state and stop walking at the endpoint in the main corridor. The black line shows the trajectory of the person calculated using a stochastic model, that is, position estimation using sensor fusion, whereas the red line is the predicted trajectory using the learning to prediction model. As we see in the results that the amount of drift in the sensor reading is greatly reduced after tuning prediction algorithms using artificial neural networks. In scenario 1, illustrated in Figure 21, the starting point is (12,2) and the endpoint is (1,24) computed using traditional Kalman filter with respect to defined reference point (0,0). Similarly, in the case of learning to Kalman filter model, the accuracy of the indoor system is improved in which the starting point and the ending point are (13,5.5) and (−2,24) receptively. All the computed coordinates are mapped according to the defined reference point (0,0).

Similarly in scenario 2, presented in Figure 22, we compute the result of the conventional Kalman filter with (3,6) as a starting point, and the ending point is mapped as −3 as x-coordinate and 24 as y-coordinate with (0,0) reference point. For the learning to prediction model, the accuracy is improved with respect to reference point (0,0), where the start point is mapped as (3,6) and the endpoint is (−3,24).

Table 6 presents the RMSE in position with the prediction model and the learning to prediction model. The results indicate that the error in position estimation is improved by 19% in the case of the learning to prediction model. Furthermore, the proposed model precise the sensor reading based on bias error correction, which results in improving the system accuracy.

As it was challenging to differentiate the result presented in Figure 19, Figure 20, Figure 21 and Figure 22. Thus, there is a need for several statistical methods to evaluate the above-presented results in a single quantifiable comparative analysis as mentioned in Equations (25)–(27). We have conducted multiple results for evaluating the performance of a traditional Kalman filter with different *R* values. Likewise, in the case of the learning to prediction module, the performance is assessed with the selected value of error factor (f). Experimental results show that Kalman filter with learning to prediction module with F=0.02 performed well as compared to other statistical measures. The best outcome for the Kalman filter with no learning module where R=20, as a result of 2.49 prediction accuracy in terms of RMSE. Similarly, the best case for the learning to prediction module is recorded with F=0.02, with a result of 2.38 prediction accuracy in terms of RMSE. Significant enhancement in the prediction accuracy of the learning to prediction module as compared to the Kalman filter with the learning module is 0.041% for the best case and 0.11% for the worst case in terms of RMSE. The statistical summary of Kalman filter for both learning and without learning module is summarized in Table 5.

## 5. Conclusions and Future Work

In this paper, we designed a learning to prediction approach, which was used to enhance the accuracy of the prediction algorithm in an indoor environment. The proposed system is a combination of learning to prediction and position estimation using a sensor fusion algorithm. In the proposed position estimation module, we used a sensor fusion technique based on Kalman filter to fuse all three sensors’ measurements in order to get the noise and drift-free orientation estimation for calculating the accurate position in indoor navigation. Likewise, we have used an ANN-based learning model in the learning to prediction module, which enhanced the accuracy of the prediction algorithm. In the designed system, we considered a scenario where the accelerometer and gyroscope sensor is affected by the external conditions, where a conventional Kalman filter failed to extract the noise-free sensor reading from the actual readings. The proposed system improved the performance of the prediction algorithm by tuning the *R* parameter. For comparative analysis, we analyzed the results using the well know statistical measures such as RMSE, MAD, and MSE. The comparative analysis suggested that the learning to prediction model in the indoor navigation system performed better with the error factor of 0.02, which resulted in a 2.38 RMSE prediction accuracy. The results indicated that the proposed learning to prediction model significantly improves the prediction accuracy and gives us the confidence to further explore the application to improve the performance of other prediction algorithms in indoor navigation systems.

## Figures and Tables

**Table 1:** Comparative analysis of Indoor localization using machine learning approaches.

Approach	Reference	Input Data	Machine Learning Algorithm	Hidden Layer	Output
Inertial Measurement Unit Data	[39]	Inertial Sensor Data (acclerometer, gyroscope, magnetometer)	Artificial Neural Network	2–4	Step Length
	[46]		Recurrent Neural Network	4	Static Detection
Radio Signal Strength	[47]	WiFi Data (Access point, nodes)	Feed-Forward Neural Network	1–3	Location
	[48]		Generative Adversarial Neural Network	3	Distance
	[49]		Artificial Neural Network	1	Location
	[50]		Radial basis Function Neural Network	1	Location
	[45]		Adaptive Neural Fuzzy Inference System	3	Distance
Channel State Information	[51]	WiFi Data (Access point, nodes)	Generalized Cross-correlation	1–2	Location
Angle of Arrival	[52]	Radio, Optical or Acoustic	Convolution Neural Network	8	Location
**Learning to Prediction**	**Proposed Solution**	**Inertial Sensor Data (acclerometer, gyroscope, magnetometer)**	**Artificial Neural Network**	**10**	**Position**

**Table 2:** Characteristic of NGIMU.

Sensor	Description	
Gyroscope	Range	±2000°/s
	Resolution	0.06°/s
	Sample Rate	400 Hz
Accelerometer	Range	±16 g
	Resolution	490 μg
	Sample Rate	400 Hz
Magnetometer	Range	±1300 μT
	Resolution	∼0.3μT
	Sample Rate	∼20 Hz

**Table 3:** Development environment for stochastic model.

Component	Description
IDE	MATLAB R2018a
Operating System	Window 10
CPU	Intel(R) Core(TM) i5-8500 CPU @ 3.00GHz
Memory	8GB
Data smoothing and
prediction algorithm	Kalman Filter
API	NGIMU

**Table 4:** Development environment for learning to prediction model.

Component	Description
IDE	MATLAB R2018a
Operating System	Window 10
CPU	Intel(R) Core(TM) i5-8500 CPU @ 3.00GHz
Memory	8GB
Artificial Neural Network	Feed Forward Backpropagation
Hidden Layer	10
output Layer	1
Input	3
Prediction algorithm	Kalman Filter

**Table 5:** Kalman filter prediction results with and with learning module.

Metric	Kalman Filter without ANN-Based Learning Module			Learning to Prediction Model		
	R = 10	R = 15	R = 20	F = 0.01	F = 0.02	F = 0.1
RMSE	2.527	2.495	2.494	2.404	2.388	2.481
MAD	0.166	0.163	0.163	0.156	0.156	0.156
MSE	6.388	6.224	6.222	5.770	5.701	6.157

**Table 6:** Position error with the prediction model and the learning to prediction model.

Experiment ID	Position Error with Prediction Model (mm)	Position Error with Learning to Prediction Model (mm)
1	0.132	0.105
2	0.115	0.099
